# RapidET: a MEMS-based platform for label-free and rapid demarcation of tumors from normal breast biopsy tissues

**DOI:** 10.1038/s41378-021-00337-z

**Published:** 2022-01-17

**Authors:** Anil Vishnu G. K., Gayatri Gogoi, Bhagaban Behera, Saeed Rila, Annapoorni Rangarajan, Hardik J. Pandya

**Affiliations:** 1 Center for BioSystems Science and Engineering, Indian Institute of Science, Bangalore, Karnataka India; 2 Department of Pathology, Assam Medical College, Dibrugarh, Assam India; 3 Department of Electronic Systems Engineering, Indian Institute of Science, Bangalore, Karnataka India; 4 Department of Molecular Reproduction, Development, and Genetics, Indian Institute of Science, Bangalore, Karnataka India; 5 Centre for Product Design and Manufacturing, Indian Institute of Science, Bangalore, Karnataka India

**Keywords:** Biosensors, Electrical and electronic engineering

## Abstract

The rapid and label-free diagnosis of malignancies in ex vivo breast biopsy tissues has significant utility in pathology laboratories and operating rooms. We report a MEMS-based platform integrated with microchips that performs phenotyping of breast biopsy tissues using electrothermal sensing. The microchip, fabricated on a silicon substrate, incorporates a platinum microheater, interdigitated electrodes (IDEs), and resistance temperature detectors (RTDs) as on-chip sensing elements. The microchips are integrated onto the platform using a slide-fit contact enabling quick replacement for biological measurements. The bulk resistivity (*ρ*_*B*_), surface resistivity (*ρ*_*S*_), and thermal conductivity (*k*) of deparaffinized and formalin-fixed paired tumor and adjacent normal breast biopsy samples from *N* = 8 patients were measured. For formalin-fixed samples, the mean *ρ*_*B*_ for tumors showed a statistically significant fold change of 4.42 (*P* = 0.014) when the tissue was heated from 25 °C to 37 °C compared to the adjacent normal tissue, which showed a fold change of 3.47. The mean *ρ*_*S*_ measurements also showed a similar trend. The mean *k* of the formalin-fixed tumor tissues was 0.309 ± 0.02 W m^−1^ K^−1^ compared to a significantly higher *k* of 0.563 ± 0.028 W m^−1^ K^−1^ for the adjacent normal tissues. A similar trend was observed in *ρ*_*B*,_ *ρ*_*S*,_ and *k* for the deparaffinized tissue samples. An analysis of a combination of *ρ*_*B*_, *ρ*_*S*_, and *k* using Fisher’s combined probability test and linear regression suggests the advantage of using all three parameters simultaneously for distinguishing tumors from adjacent normal tissues with higher statistical significance.

## Introduction

Breast cancer accounted for 15.5% of all cancer-related deaths among women worldwide in 2020 and continues to be the most commonly diagnosed form of cancer in females (23.5%)^1^. The 5-year survival rates vary significantly across cancer type and the stage of the disease at diagnosis. Breast cancer has a comparatively good survival rate and prognosis if diagnosed early and staged accurately^2^. Current cancer diagnostic methods encompass laboratory tests such as blood tests, complete blood count, and urine analysis; diagnostic imaging such as transmission, reflection, and emission imaging; and invasive tumor biopsies such as endoscopic, skin, bone marrow, fine needle aspiration, and excisional or incisional biopsy^3^. The conventional diagnosis of breast cancer involves histological analysis using hematoxylin and eosin (H&E) staining followed by immunohistochemical analysis for key biomarkers, namely, estrogen receptor (ER), progesterone receptor (PR), and human epidermal growth factor receptor 2 (HER2) staining for morphometric analysis from permanent tissue sections^4^. However, with respect to surgical margin assessment within the operating room (OR), the standard technique is frozen section examination, a pathological laboratory procedure for a comparatively faster microscopic examination of excised tissue^5–7^. However, this process involves sending the biopsy tissue out of the OR and into the pathology labs, with the analysis time for each sample ranging between 30 min and 2 h. To assess a conservative margin around the tumor, this must be carried out on multiple samples around the suspected lesion site and the surrounding lymph nodes^8^. This adds to the time of diagnosis as well as the surgery. Therefore, tools that can objectively assess the inherent physical properties of incoming biopsy tissues in pathology laboratories and quickly delineate between tumor and normal tissues have significant value in improving the diagnostic results of routine examinations^9^.

The biophysical properties of breast tissues, known to change as tumors progress, could serve as a label-free marker for delineating between tumors and adjacent normal tissues^10^. These changes are directed by remodeling the extracellular matrix (ECM) through matrix stiffening and cross-linking, collagen deposition, associated cell softening, and the induction of new vasculatures leading toward the tumor^11–13^. At the macroscale, these transformations are reflected as changes in the physical properties of the tissue, such as its electrical, thermal, and mechanical response^14–17^. Surgeons examine tissue stiffness by palpating it to assess the location of the tumor^18^. At the core of the tumor, there is a discernible difference in stiffness compared to the surrounding normal regions. This is not as pronounced at the margins, where a gradual increase in stiffness occurs when one moves from normal to tumor tissue^19,20^. This is one of the many factors making margin assessment an important clinical challenge^21^. Measurement of the electrical and thermal properties of the tissue at this margin, such as its electrical resistivity and thermal conductivity, could provide a more objective assessment for delineation. In addition, breast tumors are characterized by enhanced anisotropy, largely caused by matrix remodeling^22^. Recently, the quantification of this anisotropy has emerged as a novel marker to assess tumorigenesis and malignancy^23,24^. The capability to measure the surface and bulk physical properties, such as electrical resistivity^25^, thermal conductivity^26^, thermal diffusivity^27^, and mechanical stiffness^18,23^, from biopsy tissue would help to assess this anisotropy. This would require multidirectional probing and measurements at the millimeter scale from the sample tissue, often extracted from a standard 2–5-mm biopsy needle.

Microelectromechanical systems (MEMS), consisting of electrical and mechanical components that function as sensors or actuators, offer an exciting avenue for the manipulations at the millimeter and micrometer scales that are necessary for characterizing biopsy tissues. MEMS-based sensors and actuators, developed on substrates such as silicon, glass, and flexible materials, have been used to develop several biomedical systems covering a broad spectrum of applications, such as energy harvesting, clinical diagnostics, physiologically integrated devices, and gadgets for wellness monitoring^28–31^.

MEMS-based devices have been used for breast cancer diagnosis from ex vivo tissues^25,32,33^. However, most MEMS technology applications in breast cancer diagnosis have been at the single-cell and 2D cell culture scales using microfluidic and microcantilever-based devices^34^. Microfluidics-based technologies for breast cancer diagnosis from ex vivo samples have focused on the isolation of circulating tumor cells from blood samples, on-chip molecular diagnostics such as polymerase chain reaction (PCR) and immunocytochemistry (ICC), and the development of high-throughput drug screening platforms using cancer cells cultured from either cell lines or patient samples^35^. Tissue-level applications have been limited to 3D coculture models using spheroids and organoids to understand drug responses and cancer-specific phenotypes such as cell migration. These technologies offer a better approximation of the cellular environment than conventional cell culture systems by allowing more precise control of the culture environment. On-chip drug screening and cell sorting systems based on electrical sensing and mechanical stiffness assessment have also been developed to analyze cell cultures at a single-cell level^36,37^. However, a majority of these technologies have been limited to the laboratory scale owing to challenges in clinical translation, such as the complex nature of bulk patient samples (both tissue and blood), the matrix effect, and the need for elaborate setups such as injection/peristaltic pumps and high-speed imaging systems to perform the assays. Piezoresistive microcantilevers and piezoelectric micromachined ultrasound transducers (PMUTs) are another class of MEMS devices developed for ex vivo breast tissue characterization that assesses the mechanical stiffness, acoustic impedance, vascular density, and viscoelastic parameters of the tissue^38,39^. Such measurements have been carried out with single cells, tissue sections, and whole tissues and have shown promising results with good sensitivity to demarcate tumors from normal tissues^40^. Recently, such sensors have been integrated with custom microneedles for guided tissue targeting in the surgical resection of cancers^41^. Although such devices offer good sensitivity, the major disadvantage is the complex design of the sensor, often involving multiple process steps and the need for reliable packaging to ensure robustness and ease of handling. Other MEMS-based technologies that have been developed employ thin-film metal electrodes on silicon or glass substrates for electric cell-substrate impedance spectroscopy (ECIS), surface acoustic wave (SAW) biosensors for immunosensing of biomarkers from liquid biopsies, and microscale thermocouple probes on silicon-nitride microcantilevers to study the electrical and thermal properties of cancer cell cultures^36,42–45^. These technologies have shown promising and sensitive results with 2D cell culture and single-cell systems but are limited in their application for bulk tissue characterization owing to the nature of the sensor design and the lack of system-level setups for manipulating larger-sized tissues. The system-level challenges in integrating such devices into compact systems that probe ex vivo tissues remain less explored. The systems reported thus far are limited by sample size constraints, the requirement of elaborate optics and sample preparation, or the need for skilled personnel for intricate operation.

**Fig. 1:**
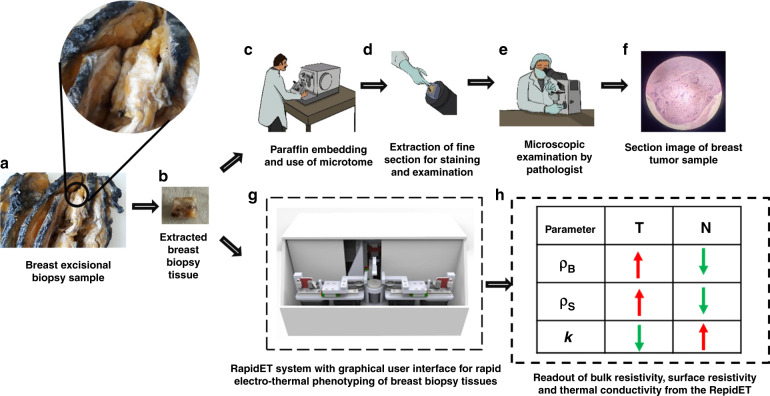
Summary of the workflow of the RapidET system for rapid label-free phenotyping of breast biopsy tissue. **a** Extraction of breast biopsy tissue from breast excisional biopsy sample, **b** photograph of extracted breast biopsy tissue, **c** frozen section preparation from the biopsy tissue using a microtome in the pathology laboratory, **d** extraction of fine section for staining and examination, **e** examination of the morphology of the tissue under the microscope by the pathologist, **f** image of stained tumor tissue section under the microscope, **g** schematic of the RapidET system for rapid electrothermal phenotyping, and **h** the summary of resistivity and thermal conductivity readout from the system for delineating between normal and tumor tissue

In this work, we report the development of technology, the RapidET system, for quick measurement of the surface and bulk electrical and thermal properties of ex vivo breast biopsy tissues to delineate tumors from adjacent normal tissues. The system integrates MEMS-based electrothermal sensors on a microchip, mechatronic actuation systems, and an application with a graphical user interface for control and acquisition. The RapidET system quantifies the surface resistivity (*ρ*_*S*_), bulk resistivity (*ρ*_*B*_), and thermal conductivity (*k*) of the sample. Measurements were performed on two types of routinely processed breast biopsy tissues found in clinical practice, namely, deparaffinized formalin-fixed paraffin-embedded (FFPE) tissues and formalin-fixed fresh tissues. The schematic of the workflow is summarized in Fig. 1. Prior to electrothermal characterization using the RapidET system, the samples were diagnosed as normal or tumor by a pathologist.

## Results

### Tumor samples have significantly higher bulk and surface resistivity at higher tissue temperatures than adjacent normal tissue

**Fig. 2:**
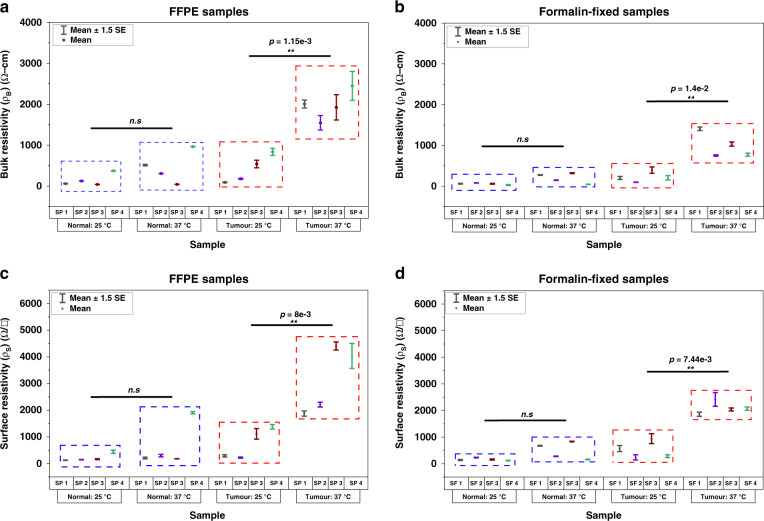
Temperature-dependent bulk and surface resistivity measurements using the RapidET system. **a** Bulk resistivity measurements from the deparaffinized normal and tumor FFPE samples at two different tissue temperatures of 25 °C and 37 °C, (**b**) bulk resistivity measurements from the formalin-fixed normal and tumor tissue samples, and (**c**) and (**d**) surface resistivity measurements from the deparaffinized FFPE and formalin-fixed normal and tumor samples at the two tissue temperatures

Bulk and surface resistivity measurements as a function of tissue temperature were performed using the RapidET system on paired (tumor and adjacent normal) deparaffinized (sample notation: “SP”) and formalin-fixed (sample notation: “SF”) breast biopsy tissues. The microheater in the microchip attached to indenter subsystem 1 (IS1) was used to externally heat the tissue sample loaded on the system. The bulk resistivity measurements were performed across each pair of interdigitated electrodes on the microchips attached to IS1 and IS2. The surface resistivity measurements were captured from the interdigitated electrodes on each microchip attached to the indenters. The tissue was heated at increments of 3 °C from 25 to 37 °C, and the measured bulk and surface resistivity values at 25 °C and 37 °C are plotted in Fig. 2 for each pair of tumor and adjacent normal tissues. Collagen fibers, which constitute a major portion of the ECM, are known to be thermally unstable beyond 37 °C^46,47^.

Accordingly, the samples were not heated beyond 37 °C for the electrothermal analysis. The deparaffinized adjacent normal tissues showed a mean bulk resistivity (*ρ*_*B*_) of 148.42 ± 76.44 Ω-cm at 25 °C and 456.09 ± 194.63 Ω-cm at 37 °C (Fig. 2a), indicating a mean increase of 3.07 times for the 12 °C rise in tissue temperature. A paired two-tailed Student’s *t* test indicated that this change was not statistically significant (*P* = 0.103*)*. The deparaffinized tumor samples showed a mean bulk resistivity (*ρ*_*B*_) of 411.25 ± 172.03 Ω-cm at 25 °C and 1980.87 ± 185 Ω-cm at 37 °C (Fig. 2a), with a mean increase of 4.82 times. This change in *ρ*_*B*_ for the deparaffinized tumor tissues was found to be statistically significant (*P* = 0.0012*)*. The *ρ*_*B*_ measured from formalin-fixed adjacent normal tissues had a mean value of 56.39 ± 10.4 Ω-cm at 25 °C and 195.70 ± 62.19 Ω-cm at 37 °C with a fold change of 3.47 (Fig. 2b). This change was, however, not found to be statistically significant (*P* = 0.0957). For the formalin-fixed tumor samples, the mean *ρ*_*B*_ was 224.125 ± 61.72 Ω-cm at 25 °C and 991.4 ± 152.92 Ω-cm at 37 °C (Fig. 2b). The increase in *ρ*_*B*_ (fold change = 4.42) was found to be statistically significant with *P* = 0.014. It should be noted that the mean *ρ*_*B*_ for both the adjacent normal and tumor tissues (at both the reported temperatures) for the formalin-fixed samples was lower than that for the deparaffinized samples.

The mean surface resistivity, *ρ*_*S*_, also showed a similar trend for both the deparaffinized (#SP) and formalin-fixed (#SF) tumor and adjacent normal samples from each patient (Fig. 2c, d). While the trend was similar, the mean surface resistivity was higher than the mean bulk resistivity for all the cases. This could be because the *ρ*_*S*_ measurements are performed along one surface of the tissue rather than through the bulk, limiting the cross-sectional area available for current flow and thus leading to higher measured resistance. Deparaffinized adjacent normal tissues had a mean *ρ*_*S*_ of 221.24 ± 74.71 Ω/□ at 25 °C and 648.2 ± 419.44 Ω/□ at 37 °C, resulting in a fold change of 2.93. This change was not statistically significant (*P* = 0.305), likely due to the high variability in the measured *ρ*_*S*_ at 37 °C. The mean *ρ*_*S*_ of the deparaffinized tumor tissues were 753.05 ± 292.05 Ω/□ and 3131.88 ± 638.18 Ω/□ at 25 and 37 °C, respectively. This fold change of 4.16 in *ρ*_*S*_ for the deparaffined tumor tissues was observed to be statistically significant (paired two-tailed Student’s *t* test) with *P* = 0.008. Following the trend of *ρ*_*B*_, the mean *ρ*_*S*_ values for the formalin-fixed tumor and adjacent normal tissues were also found to be lower than those observed for the deparaffinized tissues. The formalin-fixed adjacent normal tissues had mean *ρ*_*S*_ values of 160.5 ± 24.25 Ω/□ and 484.97 ± 159.8 Ω/□ at 25 °C and 37 °C, and the values for the tumor samples were 507.08 ± 162.19 Ω/□ and 2095.07 ± 116.83 Ω/□ at the corresponding temperatures, respectively. The change in *ρ*_*S*_ was found to be statistically significant only for the tumor tissues (fold change of 4.13 with *P* = 0.00744).

### Ex vivo tumor tissues exhibit lower thermal conductivity than normal tissues

**Fig. 3:**
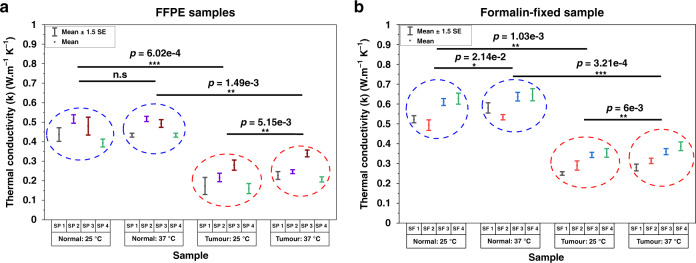
Thermal conductivity measurement using the RapidET system. **a** Thermal conductivity (*k*) of normal and tumor FFPE samples measured at 25 °C and 37 °C and **b** thermal conductivity (*k*) of formalin-fixed normal and tumor samples

The resistance temperature devices (RTDs) on each sensor were primarily used to measure the thermal conductivity of the sample under testing. While the microheater in the microchip connected to IS1 was the active source of heat for the tissue, the microheater on the chip connected to IS2 acted as an additional RTD to measure the transmitted heat through the tissue. All the RTDs were first characterized and calibrated to obtain their resistance versus temperature profile (Supplementary Fig. S1). The temperature coefficient of resistance for the fabricated Pt microheater was found to be 2.2e-3 °C^−1^. Figure 3 shows the measured thermal conductivity, *k*, for the deparaffinized (Fig. 3a) and formalin-fixed (Fig. 3b) tumor and adjacent normal tissues at 25 °C and 37 °C. The deparaffinized adjacent normal tissue had mean *k* values of 0.456 ± 0.023 W m^−1^ K^−1^ and 0.47 ± 0.018 W m^−1^ K^−1^ at 25 and 37 °C, respectively. The deparaffinized tumor tissues had mean *k* values of 0.207 ± 0.023 W m^−1^ K^−1^ and 0.255 ± 0.0255 W m^−1^ K^−1^ at these temperatures. For the formalin-fixed samples, the adjacent normal tissue showed a mean *k* of 0.563 ± 0.028 W m^−1^ K^−1^ and 0.599 ± 0.022 W m^−1^ K^−1^ at 25 and 37 °C, respectively. Mean *k* values of 0.309 ± 0.02 W m^−1^ K^−1^ and 0.335 ± 0.0206 W m^−1^ K^−1^ were observed in the formalin-fixed tumor tissues at the corresponding temperatures, respectively. A paired two-tailed Student’s *t* test was applied to assess statistically significant differences in the measured *k* (in the normal and tumor groups) between the two tissue temperatures (25 °C and 37 °C). An unpaired two-tailed Student’s *t* test with Welch correction (for unequal variance) was employed to assess the level of statistical significance between the tumor and normal groups at the two temperature points. The *k* values of deparaffinized adjacent normal tissues did not show a significant difference between 25 and 37 °C (*P* = 0.294). However, the *k* values of deparaffinized tumor tissues, formalin-fixed adjacent normal tissues, and formalin-fixed tumor tissues were found to be significantly different at 25 °C and 37 °C with *P* = 0.00515, *P* = 0.0214, and *P* = 0.006, respectively. The tumor group showed a lower mean *k* than the adjacent normal group for both sample types and at the two different temperatures (deparaffinized samples: *P* = 0.000602 (25 °C), *P* = 0.00149 (37 °C); formalin-fixed samples: *P* = 0.00103 (25 °C) and *P* = 0.000321 (37 °C)). The level of statistical significance was, on average, an order of magnitude higher when the tumor and normal groups were compared at each temperature as opposed to when the normal and tumor groups are individually compared across the two temperature points. The measured mean *ρ*_*B*_, *ρ*_*S*_, and *k* values from the deparaffinized and formalin-fixed samples are summarized in Table 1.

**Table 1:** The measured mean bulk resistivity (*ρ*_*B*_), surface resistivity (*ρ*_*S*_), and thermal conductivity (*k*)

		Normal (N)			Tumor (T)			T/N	
		25 °C	37 °C	N_(37)_/N_(25)_	25 °C	37 °C	T_(37)_/T_(25)_	T_(25)_/N_(25)_	T_(37)_/N_(37)_
Mean bulk resistivity (*ρ*_*B*_) (Ω-cm)	Deparaffinized samples	148.42 ± 76.44	456.09 ± 194.63	3.07	411.25 ± 172.03	1980.87 ± 185.002	4.82	2.77	4.34
	Formalin-fixed samples	56.39 ± 10.4	195.70 ± 62.19	3.47	224.125 ± 61.72	991.4 ± 152.92	4.42	3.97	5.07
Mean surface resistivity (*ρ*_*S*_) (Ω/□)	Deparaffinized samples	221.24 ± 74.71	648.2 ± 419.44	2.93	753.05 ± 292.05	3131.88 ± 638.18	4.16	3.4	4.83
	Formalin-fixed samples	160.5 ± 24.25	484.97 ± 159.81	3.02	507.08 ± 162.19	2095.07 ± 116.83	4.13	3.16	4.32
Mean thermal conductivity (*k*) (W m^−1^ K^−1^)	Deparaffinized samples	0.456 ± 0.023	0.47 ± 0.018	1.03	0.207 ± 0.023	0.255 ± 0.0255	1.23	0.454	0.543
	Formalin-fixed samples	0.563 ± 0.028	0.599 ± 0.022	1.06	0.309 ± 0.02	0.335 ± 0.0206	1.08	0.55	0.56

## Discussion

A higher resistivity for tumor tissue than for normal tissue is consistent with the results for ex vivo breast biopsy tissues shown by Morimoto et al., Massalska et al., and Jossinet et al., where breast cancer tissues showed a higher resistance than healthy mammary gland tissue^48–50^. The higher resistivity of tumor tissue is due to matrix remodeling and increased disorder in the tumor microenvironment compared to the normal microenvironment^11,12,22,23^. The electrical equivalent circuit of biological tissue can be considered as a combination of extracellular resistance, cell membrane resistance and capacitance, and intracellular resistance^51^. When measuring the electrical resistivity of a tissue sample, extracellular resistance becomes the key determinant, as the capacitance of the cell membrane blocks direct current (DC) flows through the cells^52^. Thus, the effective resistivity of the tissue is determined by the nature and volume fraction of the ECM, which is mainly composed of type I and III collagen^53^. While this is a basic electric model for any tissue, certain physiological changes that occur in tumor tissues lead to the differences in measured resistivity between tumor and normal tissues. During tumor initiation and progression in breast tissues, the collagen fibers in the ECM have been reported to become degraded and fragmented^54–57^. This leads to fibers with a shorter length within the ECM of the tumor, resulting in a higher tissue bulk resistivity, similar to the phenomenon observed in composite materials with conductive fillers^58,59^.

In addition, the surface resistivity measurement helps to indirectly characterize the surface irregularities and conformability of the sample to the microchip surface, as these have direct effects on the measured *ρ*_*S*_^60^. A higher value of *ρ*_*S*_ indicates higher surface irregularities and poorer conformability to the microchip surface in the sample. Due to the higher volume fraction of the remodeled ECM, the tumor tissues are known to be stiffer than the adjacent normal tissues^61^. The enhanced stiffness makes the tumor samples less conformal to the microchip surface than the adjacent normal tissues, thereby leading to a higher *ρ*_*S*_. The higher disorder and entropy in the tumor lead to a higher increase in bulk and surface resistivity with temperature than in the adjacent normal tissue. The temperature-sensitive nature of collagen fibers observed in solution form also contributes to the increased resistivity^46,47^. Since the tumor tissues have a higher volume fraction of these fibers, with more fragmented fibers, the increase in resistivity (surface and bulk) is more pronounced in tumor tissues than normal tissues.

The tumor samples were observed to have significantly higher bulk (*ρ*_*B*_) and surface resistivity (*ρ*_*S*_) at 37 °C than at 25 °C. This considerable increase in resistivity with temperature was not observed for the normal tissues for either of the sample types studied (formalin-fixed and deparaffinized). Even though the tumor and adjacent normal tissue samples were both observed to have significantly different *ρ*_*B*_ and *ρ*_*S*_ at 37 °C, which can also serve as a basis for delineation, we believe that the significantly higher *ρ*_*B*_ and *ρ*_*S*_ observed for the tumor tissue at 37 °C compared to 25 °C is of greater practical utility, as it can potentially eliminate the need for paired adjacent normal tissue.

The idea of the study is to move toward a scenario in which the surface and bulk electrothermal characterization of the biopsy sample would help classify it as a “tumor” or “normal”. From a translational perspective, since obtaining adjacent normal tissue along with a suspected tumor sample has practical limitations, heating the same tumor tissue at two temperatures (25 °C and 37 °C) and comparing the bulk and surface resistivity would serve as a novel biomarker and reduce the dependence on acquiring an adjacent normal tissue to classify the biopsy. This bimodal approach of measuring the bulk and surface resistivity of the sample at elevated temperatures could thus be used as a basis for classifying the sample as a tumor or normal tissue.

In regard to the differences in measurements between formalin-fixed and deparaffinized samples, our results show that the mean bulk and surface resistivity values of formalin-fixed (normal and tumor) tissues are lower than the values for the deparaffinized (normal and tumor) tissues at both 25 °C and 37 °C. However, the trend of a significantly higher increase in the surface and bulk resistivity in tumors with tissue temperature compared to the adjacent normal tissue is consistent across both types of samples studied. This is primarily because the process of formalin fixation is common across both sample types, with an additional step of paraffin embedding performed to prepare FFPE samples. This embedded paraffin is then removed through the deparaffinization protocol to prepare the samples. Although formalin fixation removes free water in cells and tissues, it preserves the tissue organization, the microarchitecture of extracellular fibers, proteins, and lipids, and thus the structural and compositional differences between ex vivo tumor and normal tissues^62^. The additional steps followed for FFPE block preparation after formalin fixation, namely, gradual dehydration and high-temperature paraffin embedding, are known to quench a small subset of proteins with minimal tissue damage^63,64^. The samples are then rehydrated after paraffin removal during the deparaffinization process. It has also been reported that the existing laboratory protocols for deparaffinizing FFPE tissue blocks still leave behind traces of paraffin-embedded in the tissue^65^. Such paraffin left behind in the tissue has been found to affect the contrast and intensity of the immunohistochemical staining while not detrimentally affecting the proteins and other constituents probed by the technique^66^. Paraffin wax has a very high electrical resistivity (10^12^ Ωm–10^17^ Ωm). The bulk and surface resistivity values observed in our study are on the order of 10^3^ Ωm, suggesting that any traces of paraffin retained in the deparaffinized sample had only a negligible effect on the measurements. Thus, the additional steps followed during FFPE sample preparation and subsequent deparaffinization might have contributed to the samples’ higher mean bulk and surface resistivity while not significantly altering the inherent differences between tumor and normal tissue, as evidenced by the results observed.

Breast tumor tissues have been reported to have higher thermal conductivity (~0.62 W m^−1^ K^−1^) than normal breast tissue (~0.48 W m^−1^ K^−1^) in in vivo measurements from fresh tissues^26,67,68^. However, ex vivo measurements have indicated a lower thermal conductivity for tumors than for normal tissues^69,70^. Both the cellular and extracellular composition of the tissue contribute to the bulk thermal conductivity. Collagen and other fibers of the ECM, blood vessels, and water content in the extracellular space are some of the key elements contributing to thermal transport through the matrix. In the intracellular space, the amount of proteins, intracellular water content, glycogen, lipids, and nuclear–cytoplasm ratio are the key determinants of thermal conductivity^71^. In the in vivo scenario, the tumor tissue, a site of intense vasculature, metabolic activity, and water content, has enhanced blood perfusion rates, contributing to the increased thermal conductivity compared to that of structured normal tissues^72–74^. Thermal conduction in the in vivo tumor tissues is thus dominated by the extracellular milieu owing to the high thermal conductivity of blood and water (greater than 0.5 W m^−1^ K^−1^)^75^. In ex vivo formalin-fixed and deparaffinized tissues, there is no internal metabolic activity or heat dissipation from blood perfusion.

In addition, the process of formalin fixation removes the free water content in the sample while preserving tissue constituents such as proteins, lipids, and collagens^62,76^. Collagens, which are structural proteins of the matrix, and other intracellular proteins, have a lower thermal conductivity in the range of 0.2 W m^−1^ K^−1^–0.3 W m^−1^ K^−1^ (see ref. ^77^). Likewise, lipids have thermal conductivity in the range of 0.1 Wm^−1^ K^−1^–0.3 Wm^−1^ K^−1^ (see ref. ^78^). With the free water content in the tissue removed through formalin fixation, the majority of the thermal conduction occurs through collagens, lipids, and proteins in the cellular and extracellular space. Given that tumor tissues have higher disorder, leakier vasculature, and higher aqueous content, the effects of formalin fixation are thus expected to be more pronounced in tumors than in normal tissues.

The intracellular changes during tumor initiation and progression also contribute to the lower thermal conductivity. Tumor cells are characterized by an increased number of free ribosomes and polysomes, which lead to increased protein production required for cell growth^71^. Tumor cells are also characterized by a higher nuclear–cytoplasm ratio and an increased number of nucleoli (which mainly consist of RNA, DNA, and proteins), which consequently reduces the water content in the cytoplasm^79^. In addition, tumor cells are known to contain a reduced amount of glycogen, which concurrently increases the lipid content in the cells^78^. These changes, occurring at both the extracellular and intracellular levels, lead to a lower thermal conductivity in tumors than in adjacent normal tissues, as observed in the measurements from deparaffinized and formalin-fixed samples. Thus, characterizing the thermal conductivity of ex vivo biopsy tissues provides a means of classifying them into tumor or normal tissue sample.

**Fig. 4:**
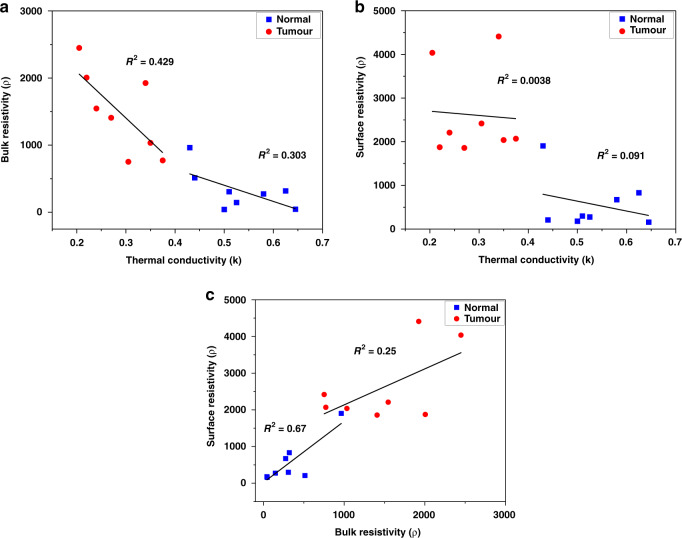
Regression analysis for the combination of measurement parameters. **a** Scatter plot showing the correlation between bulk resistivity and thermal conductivity for the measured samples. The plot demonstrates a clear demarcation between tumor and normal samples. **b** Correlation plot between surface resistivity and thermal conductivity and **c** correlation plot between surface and bulk resistivity

To understand whether a combination of measurement parameters (viz. bulk resistivity (*ρ*_*B*_), surface resistivity (*ρ*_*S*_), and thermal conductivity (*k*)) can help to better delineate tumors from normal tissues, the Fisher’s combined probability test was performed to obtain p values for different combinations of the parameters. Statistical tests were performed to evaluate whether these parameters differed significantly between normal tissues at two different temperatures, between tumor tissues at the two different temperatures, and between tumor and normal tissues at each of the two temperatures. The delineation was most significant when a combination of all three parameters measured at 37 °C was employed to differentiate between tumor and normal tissues, irrespective of using deparaffinized samples (*P* = 6.74e-6) or formalin-fixed samples (*P* = 1.95e-7). While the difference in the measured parameters across the two reported temperatures of 25 °C and 37 °C continued to be nonsignificant (*P* = 1.54e-1 in case of deparaffinized) or of low significance (*P* = 1.24e-2 in case of formalin-fixed) for normal tissue, a combination of all three parameters was able to capture the difference in tumor tissue with the most significance in both deparaffinized (*P* = 7.6e-6), and formalin-fixed samples (7.3e-5). The system takes 14 ± 2 min to complete the combined analysis of a sample and give the result. This indicates the advantage of using a combination of measurement parameters to improve the delineation of tumors from normal tissues. Furthermore, to assess the independent nature of these parameters, pairwise linear regression was performed on all the normal and tumor samples. These results are summarized in Fig. 4a–c. A clear separation between the tumor (marked red) and normal (marked blue) samples was observed on the regression plot. The low R-square value obtained for the regression fits for the pairwise analysis confirms that the parameters are independent. *ρ*_*S*_ and *ρ*_*B*_ were the least independent variables, with an R-square of 0.25 for tumor samples and 0.67 for normal tissue samples. In comparison, *ρ*_*S*_ and *k* were the most independent pair, with an R-square of 0.0038 for tumors and 0.091 for normal tissues. The linear regression analysis correlated with the findings from the combined probability tests. The results of the statistical analysis for individual parameters and combinations of parameters are summarized in Table 2.

**Table 2:** Statistical analysis of measurement parameters and their combination for tumor and adjacent normal samples (statistical significance cutoff of *P* < 5e-2)

	Deparaffinized samples				Formalin-fixed samples			
Parameters	N_(37)_ and N_(25)_	T_(37)_ and T_(25)_	T_(25)_ and N_(25)_	T_(37)_ and N_(37)_	N_(37)_ and N_(25)_	T_(37)_ and T_(25)_	T_(25)_ and N_(25)_	T_(37)_ and N_(37)_
*ρ*_*B*_	1.03e-1 (ns)	1.15e-3 (**)	2.33e-1 (ns)	1.3e-3 (**)	9.57e-2 (ns)	1.4e-2 (*)	7e-2 (ns)	8.7e-3 (**)
*ρ*_*S*_	3.05e-1 (ns)	8e-3 (**)	1.65e-1 (ns)	2.15e-2 (*)	1.43e-1 (ns)	7.44e-3 (**)	1.21e-1 (ns)	2.89e-4 (***)
*k*	2.94e-1 (ns)	5.15e-3 (**)	6.02e-4 (***)	1.49e-3 (**)	2.14e-2 (*)	6e-3 (**)	1.03e-3 (**)	3.21e-4 (***)
*ρ*_*B*_ and *ρ*_*S*_	1.40e-1 (ns)	1.16e-4 (***)	1.64e-1 (ns)	3.21e-4 (***)	7.24e-2 (ns)	1.1e-3 (**)	4.94e-2 (*)	3.51e-5 (***)
*ρ*_*B*_ and *k*	1.36e-1 (ns)	7.72e-5 (***)	1.4e-3 (**)	2.74e-5 (***)	1.47e-2 (*)	8.72e-4 (***)	7.6e-4 (***)	3.85e-5 (***)
*ρ*_*S*_ and *k*	3.06e-1 (ns)	4.57e-4 (***)	1e-3 (**)	3.64e-4 (***)	2.08e-2 (*)	4.92e-4 (***)	1.2e-3 (**)	1.6e-6 (***)
*ρ*_*B*,_ *ρ*_*S*_, and *k*	1.54e-1 (ns)	7.6e-6 (***)	1.6e-3 (**)	6.74e-6 (***)	1.24e-2 (*)	7.3e-5 (***)	7.12e-4 (***)	1.95e-7 (***)

*0.01 < *p* < 0.05, **0.001 < *p* < 0.01, ***p < 0.001

In conclusion, a microchip integrated with a heater and electrical and thermal sensors for the label-free phenotyping of breast biopsy tissues has been designed and fabricated. The RapidET system, designed with a top-down approach that integrates the microchip, mechanical actuators, electronic modules, and intuitive interface application has been developed. Measurements from deparaffinized and formalin-fixed *N* = 8 paired tumor and adjacent normal breast biopsy tissues showed a significant increase in bulk resistivity (*ρ*_*B*_) and surface resistivity (*ρ*_*S*_) in tumor samples from 25 to 37 °C. Such a significant increase was not observed in the adjacent normal tissues in either type of samples studied, providing a basis for delineation. A significantly lower thermal conductivity (*k*) was observed for tumors than for the adjacent normal tissues at 25 °C and 37 °C. An analysis with a combination of all three parameters (*ρ*_*B*_, *ρ*_*S*_, and *k*) delineated tumor from normal tissues with higher statistical significance in deparaffinized (*P* = 6.74e-6) and formalin-fixed samples (*P* = 1.95e-7). The process of tumorigenesis and tumor progression alters the microarchitecture of both the intracellular and extracellular space^80,81^. Thus, to accurately differentiate between tumors and adjacent normal tissues, it is important to characterize both regions of the tissue. The bulk and surface resistivity measurements help characterize the ECM and collagen fibers through direct current electrical transport. Thermal conductivity measurements measure the thermal transport contributed by intracellular proteins, lipids, and ECM. Thus, combining thermal conductivity measurements, electrical resistivity measurements, and their temperature-dependent variations provides better insights into the changes occuring at both the cellular and extracellular levels through their bulk values, helping to better classify a sample as a tumor or normal. This is also reflected in the lower *P* value obtained when all three parameters were combined to differentiate the samples into tumor and adjacent normal tissues.

The measurement of each sample takes 14 ± 2 min, paving the way for potential application in a clinical setting. The RapidET system has been designed for characterizing ex vivo breast biopsy tissues. The microchip, indenters, and tissue holder are designed to probe an ex vivo sample and measure its surface and bulk electrical and thermal properties. The dimensions of the system (205 × 310 × 165 mm (L × B × H)) restrict its use in an in vivo application. Supplementary Fig. S2 shows the engineering drawings of the system with the dimensions. However, the modalities (electrical and thermal) discussed in the study and methods for statistical analysis could potentially be adopted when designing a system for in vivo studies. Based on the literature, we believe that the in vivo measurements would only reduce the baseline values of the resistivity owing to the enhanced conductivity provided by blood, water, and other body fluids in the in vivo scenario. Regarding the thermal conductivity, the in vivo measurements might show a higher thermal conductivity for tumors than the adjacent normal tissues due to leaky vasculature observed in tumors compared to an ordered arrangement in the normal tissues, which can enhance thermal conduction through the blood perfusion term in the Pennes bioheat equation. We envisage studying these aspects in the next phase by developing a probe integrated with a microchip and after obtaining ethics approval for in vivo experiments.

## Materials and methods

### Microchip for electrothermal phenotyping

**Fig. 5:**
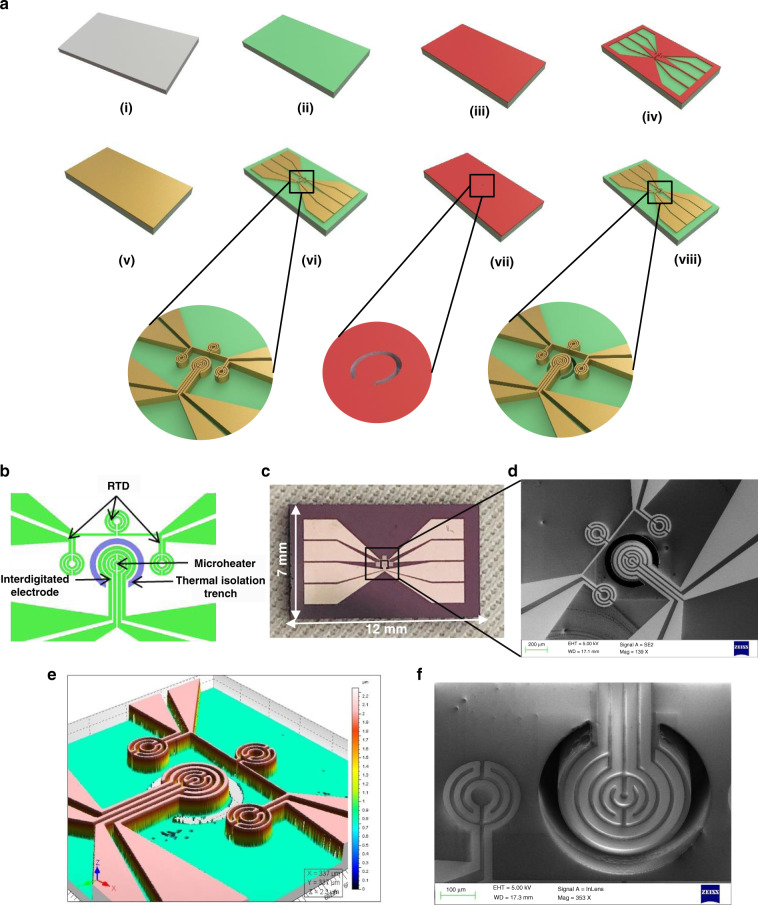
Microchip for electrothermal phenotyping: design and fabrication. **a** Process flow for the fabrication of the microchip for electrothermal phenotyping of the breast biopsy tissues, **b** design of the microchip showing the different functional components used for sensing, **c** optical photograph of the microchip fabricated on a silicon substrate, **d** scanning electron micrograph of the active area of the microchip, **e** optical profilometry of the active area of the sensor showing the elevated platinum film, and **f** a close-up scanning electron microscope image with a 20° tilt angle showing the thermal isolation trench

The process flow for microchip fabrication is shown in Fig. 5a. Figure 5b depicts the functional sensing elements of the microchip consisting of a platinum microheater at the center, three resistance temperature detectors (RTDs), and an interdigitated electrode (IDE) around the microheater. A thermal isolation trench separates the microheater from the RTDs. The microheater, electrodes, and resistance temperature detectors of the device were fabricated on an oxidized silicon wafer (1-μm SiO_2_ thermally grown on top of silicon). The thermal conductivity of thin-film silicon dioxide (~1.3 W m^−1 ^K^−1^), although much lower than that of silicon, is still higher than that of the formalin-fixed and deparaffinized tissue samples characterized in this study. However, when the input voltage is applied through the voltage driver circuit, the microheater reaches a stable operating temperature for each applied voltage. The temperature of the microheater heats the tissue. A thermal isolation trench of 350-μm depth around the microheater helps to limit the temperature of the isothermal regimes in the bulk silicon and to achieve a more uniform higher temperature regime around the microheater. This also reduces the effect of substrate heating on the resistance temperature detectors. A numerical simulation using COMSOL Multiphysics was performed to assess the effect of the trench, and the results are shown in Supplementary Fig. S3. Supplementary Fig. S3a and S3b show the heatmap of the thermal profile of the device with and without the trench, respectively. Supplementary Fig. S3c and S3d show 2D line plots of the temperature values along the axis of the microheater. The results show the improved uniform temperature regime around the microheater obtained in the presence of the trench and an overall lower temperature in the bulk substrate. A higher input voltage was required for the design without the trench to achieve the same temperatures as the design with the trench, suggesting higher thermal dissipation in bulk without the trench. The active region of the microchip covers a 1 × 0.5 mm area, and the overall chip dimension is 12 × 7 mm.

The microchip was fabricated using a two-mask process (Fig. 5a). (i) A 500-µm-thick single-side polished (100) silicon wafer was used as the substrate. (ii) silicon dioxide (SiO_2_) of 1-µm thickness was grown using thermal oxidation. (iii) The substrate was then coated with AZ5214E positive photoresist, followed by (iv) photolithographic exposure using the Suss MicroTec MJB4 mask aligner system and development. (v) Titanium/platinum (25 nm/190 nm) was then deposited using the TECPORT E-beam evaporator system and (vi) patterned using the lift-off technique. (vii) AZ4562 photoresist was then spin-coated, followed by UV exposure using a photomask for creating the pattern for the trench. The wafer was further developed with an MF26A developer followed by treatment with buffered hydrogen fluoride (BHF) for etching the silicon dioxide around the trench. (viii) deep reactive ion etching (DRIE) with the SPTS LPX Pegasus system was used to create the trenches for thermal isolation followed by photoresist stripping to obtain the final device. The wafer was then diced into individual microchips using an automatic dicing machine. A photograph of a single diced microchip is shown in Fig. 5c. Figure 5d shows a scanning electron microscope image of the microchip integrated with the microheater, RTDs, and IDEs. Figure 5e shows the optical profilometry image of the microchip showing the platinum film that forms the sensing layer. Figure 5f shows a close-up scanning electron microscope image of the microheater at a tilt angle of 20°_,_ clearly depicting the thermal isolation trench.

### The RapidET system: a platform for label-free phenotyping of breast biopsy tissues

The mechanical and electronic modules constitute the two key components of the RapidET system.

#### Mechanical module

**Fig. 6:**
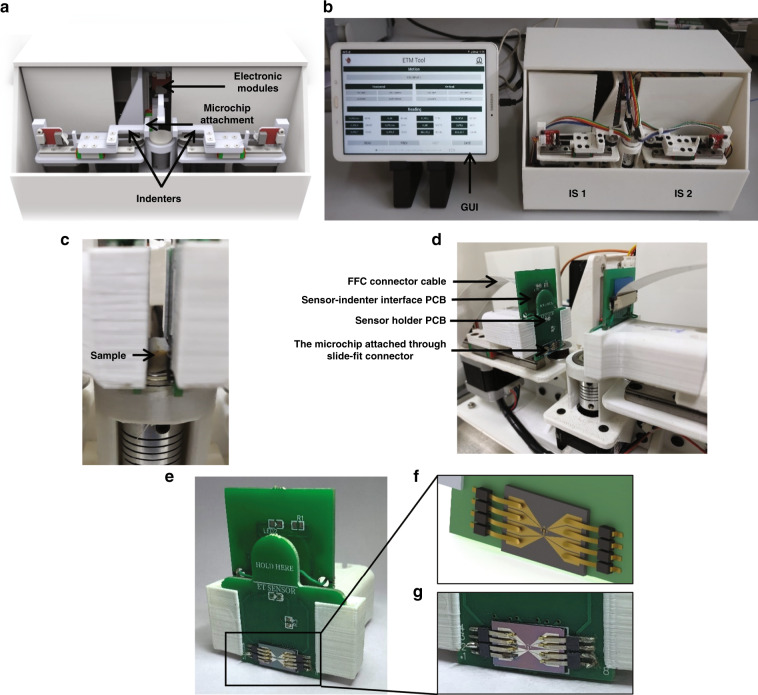
RapidET system design. **a** 3D schematic of the platform showing the various subsystems, **b** photograph of the fabricated system interfaced with a tablet PC running the graphical user interface for control and data acquisition, **c** sample loaded between the two indenter subsystems, **d** the indenter subsystem with the PCBs, microchip attached to slide-fit contacts, and FFC connector cable going into the electronic modules in the system, **e** close-up view of the microchip packaged into the sensor holder structure through slide-fit contacts, **f** and **g** rendered and actual image of the microchip connected to the slide-fit contacts, eliminating the need for wire-bonding

The system has overall dimensions of 205 × 310 × 165 mm (L × W × H). The outer casing of the system was fabricated using machined acrylic sheets, and it runs on a 230 V AC supply through a switched-mode power supply. The 3D schematic of the system is shown in Fig. 6a, and a photograph of the actual system integrated with software running on a tablet PC is shown in Fig. 6b. The sample tissue can be loaded on a removable sterilizable tissue holder, which is then placed on a fixed rotary stage. The rotary stage can rotate a full 360° with control of the degree of rotation. The rotary table is mounted directly on a NEMA 17 stepper motor. Two indenters are connected diametrically opposite to each other with the tissue holder in the middle, as shown in Fig. 6c. The indenters are placed on a linear rail with rail block MGN9C. A closed-circuit timing belt system is made using a GT2-16 tooth pulley placed directly on NEMA 17 motors and one idler pulley on the other side. A spring is used to tighten the timing belt. The combination of a timing belt, a spring, and the length between two poles is designed so that even if the indenter gets stuck somewhere during the motion, the timing belt can take a slip as a passive safety to the system and sensor module. The timing belt is attached to the indenter using a 3D-printed clamp. This arrangement constitutes the indenter subsystem (IS).

There are two indenter subsystems, IS1 and IS2, for probing the tissue from either side. IS1 and IS2 are controlled using the software running on a tablet PC. The software communicates with the electronic modules in the platform to realize the actuations of the indenters. A detachable sensor module (Fig. 6d, e) can be attached to the ends of these indenters to connect the microchips to the electronic modules for acquiring measurements from the tissue sample. This sensor module consists of a sensor holder PCB and sensor-indenter interface PCB. The microchip is attached to the sensor holder PCB via slide-fit connectors.

Figure 6f shows a schematic of the design of the slide-fit arrangement, and Fig. 6g shows a photograph of the realized mechanism. A slide-fit arrangement eliminates the need for wire-bonding during packaging, enabling easy replacement with a new microchip for each biological measurement. This design takes into account the sterilizability of the system, considering that it has to handle breast biopsy samples. The number of microchips on each wafer is sixty, and in each cycle, four such silicon wafers can be processed, thus making the overall cost of each microchip less than US$ 2.5. The overall cost of the system was computed to be US$ 480 (detailed cost is provided in Supplementary Table 1). A flexible flat cable (FFC) cable connects the sensor module to the electronics in the platform.

Supplementary Fig. S4a and S4b show the schematic of the test procedure once a tissue sample is loaded for measurement on the system.

#### Electronic module

The electronic system architecture consists of a primary and secondary microcontroller connected in a master-slave manner. The sensing and actuating lines from the microchip and the graphical user interface (GUI) are connected to the main microcontroller, while the controls for the actuation of the mechanical motions of the system are connected to the secondary microcontroller (Supplementary Fig. S5). The main microcontroller controls communications with the GUI (sends and receives soft commands from the GUI) and serves as a master for the secondary microcontroller that performs the motor control operations. A schematic of the main microcontroller is shown in Supplementary Fig. S6. Each microchip has eight logic lines for measuring the resistance values from the IDEs and RTDs. All the signal lines from the chips in the X and Y indenters are connected to a multiplexing circuit. Both microchips are connected to two 16:1 multiplexer modules. This is to facilitate the selection of any pair of logic lines from the two microchips for connecting to the resistance measurement circuit. Depending on the selection, pairs of resistances on the microchip are selected and measured using an autoranging circuit. The autoranging circuit reads the voltage value corresponding to the unknown resistance, which is then converted to the digital form by the 14-bit ADS1115 analog-to-digital converter module. The unknown resistance value is then computed from the digitized voltage value in the software and is stored and displayed on the GUI (details of the GUI are shown in Supplementary Fig. S7). The tablet with the GUI is connected to the main microcontroller via the USB channel through a universal asynchronous receiver transmit (UART) line. It can send commands for calibration, actuation of the indenters, and cycling through the measurements automatically after receiving the start measurement command from the GUI. The heating of the microheater on the microchip connected to the X-indenter is carried out by providing the required precalibrated voltage corresponding to the target temperature through the voltage driver circuit connected to the main microcontroller.

### Sample preparation

The study was performed in accordance with the ethical committee clearance obtained from Assam Medical College (Ref. No: AMC/EC/1334) and the biosafety clearance obtained from the Institutional Biosafety Committee at the Indian Institute of Science (Ref. no.: IBSC/IISc/HP/01/2019). Only a portion of the samples taken for routine examination from the patient were used for the study, and no additional samples were extracted. The patients were informed about the study through an informed consent form. Electrothermal measurements were performed on tissue samples from *N* = 8 patients. These samples were obtained from mastectomy, lumpectomy, or excisional biopsy procedures performed on the patients. Of the total *N* = 8 patients, the samples from *N* = 4 patients were preserved using formalin-fixed paraffin embedding (FFPE), and samples from *N* = 4 patients were preserved using formalin fixation using 10% formalin. The paired tumor and adjacent normal tissue blocks for the study were extracted from the FFPE and formalin-fixed samples from *N* = 8 patients by the pathologist. The samples were marked as a tumor or adjacent normal by the pathologist using histopathological analysis through hematoxylin and eosin staining. The histological nature of the tumor samples was determined to be either invasive ductal carcinoma (IDC) or fibroadenoma (representative images from the samples are shown in Supplementary Fig. S8). From each paired patient sample, *n* = 3 tumors and *n* = 3 adjacent normal tissue samples were prepared as technical repeats. Thus, a total of *n* = 48 samples consisting of *n* = 24 tumor samples and *n* = 24 adjacent normal tissue samples were prepared from the *N* = 8 patient samples.

For the FFPE samples (tumor and adjacent normal), uniform cubical blocks of dimension 5 ± 0.15 mm × 5 ± 0.23 mm × 3 ± 0.13 mm (L × B × H) were extracted from the paraffin blocks by the pathologist using a surgical knife and then deparaffinized. The FFPE samples were deparaffinized using a protocol similar to that used for immunohistochemical examination^82^. The samples were first kept in a hot air oven at 70 °C for 2 h to melt the paraffin. This step was followed by a xylene dip for 8 min to remove any remaining paraffin wax and then a serial ethanol wash with concentrations of 100%, 95%, 70%, and 50% ethanol. Each wash was performed for 5 min to remove the xylene and rehydrate the sample. After this, the samples were stored in phosphate-buffered saline (PBS) at pH 7.4 in 1.5-mL Eppendorf tubes.

The formalin-fixed samples were stored in a 10% buffered formalin solution at room temperature. From these samples, uniform cubical blocks with dimensions of 5 ± 0.15 mm × 5 ± 0.23 mm × 3 ± 0.13 mm (L × B × H) were extracted by the pathologist and stored in PBS Eppendorf tubes. The formalin fixation time for all the samples was kept uniform to prevent confounding effects.

### Model

The tissue sample can be treated as a two-component system consisting of the cells and the matrix with the effective measured resistivity given by Eq. (1)

1 $$\rho _B = \frac{{R_B.A}}{l}{\it{\Omega }}.m$$

Where *ρ*_*B*_ is the bulk resistivity of the sample, *R*_*B*_ is the measured resistance across the bulk of the tissue, A is the cross-sectional area of the outer electrode of the microchip in the RapidET system, and *l* is the thickness of the tissue sample loaded between the two indenters. The bulk resistivity of the sample loaded on the system is measured across each pair of interdigitated electrodes in the microchips connected to indenters IS1 and IS2.

The surface resistivity, *ρ*_*S*_ of the sample is measured using the interdigitated electrodes on each microchip. The *ρ*_*S*_ measurements are carried out across the surface of the tissue that is in contact with the microchip, as opposed to across the bulk of the tissue in the case of *ρ*_*B*_. For the circular and concentric design of the interdigitated electrodes in the microchip, the surface resistivity is computed using the relation Eq. (2) as discussed in Vila et al. ^83^.

2 $$\rho _S = \frac{{2\pi }}{{\ln \left( {\frac{{D_2}}{{D_1}}} \right)}}R_s{\it{\Omega }}/\square$$

where *ρ*_*S*_ is the surface resistivity, *R*_s_ is the measured surface resistance between the electrodes in the IDE structure on the microchip, *D*_2_ is the inner diameter of the outer electrode of the IDE pair, and *D*_1_ is the outer diameter of the inner electrode. For the microchip design in Fig. 2, *D*_2_ = 140 μm and *D*_1_ = 130 μm. This reduces Eq. (2) to

3 $$\rho _S = 84.8R_s{\it{\Omega }}/\square$$

Equations (1) and (3) are used to compute the bulk and surface resistivity from the measured bulk and surface resistance values at each tissue temperature. The increased disorder in the tumor tissue leads to a substantially higher resistance to current flow at higher tissue temperatures compared to the corresponding increase in normal tissues. This hypothesis is tested in the study performed with the RapidET system.

While the tissue is heated to measure the bulk and surface resistivity, its thermal conductivity value is also computed to gauge the differences in thermal transport between tumor and normal tissue. The mathematical basis for thermal transport in biological tissues is the Pennes bioheat transfer Eq. (4), which has been a standard model for understanding heat transport in tissues^84^.

4 $$\rho _vC_p\frac{{\partial T}}{{\partial t}} = \nabla \left( {k\nabla T} \right) + w_bC_b\left( {T_a - T} \right) + q_d + q_m$$

where ρ_v_ is the volumetric density of the tissue, *C*_*p*_ is the specific heat capacity of the tissue, *T* is the tissue temperature, *T*_*a*_ is the arterial temperature in the blood vessels, *w*_*b*_ is the blood perfusion rate, *C*_*b*_ is the specific heat capacity of the blood, *k* is the thermal conductivity of the tissue, *q*_*d*_ is the energy deposition rate, and *q*_*m*_ is the rate of metabolic heat generation. For ex vivo tissue, the contribution from blood perfusion and metabolic heat generation vanishes. Equation (4) reduces to Poisson’s equation under steady-state conditions given by Eq. (5)^69^.

5 $$Q = k\nabla ^2T$$

Here, *Q* is the energy supplied to heat the tissue. For the tissue sample heated from one end using the microheater in the microchip, under the assumption of uniform heating, in steady-state, the thermal conductivity, *k* can be computed using Eq. (6).

6 $$k = \frac{{Ql}}{{A\Delta T}}$$

Where *l* is the thickness of the tissue, *A* is the cross-sectional area through which the heat is transferred, and ∆*T* is the steady-state temperature gradient across the tissue; between the actively heated surface and the surface along which the transmitted heat is measured. In the RapidET system, the microheater structure in the microchip connected to IS1 is actively heated, while the same structure in the microchip connected to IS2 acts as a resistance temperature detector (RTD). The energy supplied, *Q* is computed from the voltage, and current values used to heat the microheater to the specific temperature, and the RTDs across the tissue on IS2 detect the sink temperature. From the source temperature of the microheater and the triangulated sink temperature from the RTD structures, ∆*T* is calculated. These values, when substituted into Eq. (6), give the thermal conductivity of the tissue at the specific tissue temperature *T*.

## Supplementary information

Supporting information

Supplementary Material for RapidET System
