# Supplementary material for: RapidET: a MEMS-based platform for label-free and rapid demarcation of tumors from normal breast biopsy tissues
Source: Microsyst Nanoeng. 2022 Jan 17;8:1. doi: 10.1038/s41378-021-00337-z (PMC8761751; doi:10.1038/s41378-021-00337-z)
Supplement: Supplementary file 1 — Supporting information [file 41378_2021_337_MOESM1_ESM.docx]

**SUPPLEMENTARY INFORMATION**

***for***

**RapidET: A MEMS-based platform for label-free and rapid demarcation of tumors from normal breast biopsy tissues**

Anil Vishnu G K^1^, Gayatri Gogoi^2^, Bhagaban Behera^3^, Saeed Rila^3^,

Annapoorni Rangarajan^4^ and Hardik J. Pandya^3,5^

**AFFILIATIONS**

^1^Center for BioSystems Science and Engineering, Indian Institute of Science, Bangalore.

^2^Department of Pathology, Assam Medical College, Dibrugarh.

^3^Department of Electronic Systems Engineering, Indian Institute of Science, Bangalore.

^4^Department of Molecular Reproduction, Development, and Genetics, Indian Institute of Science, Bangalore.

^5^Centre for Product Design and Manufacturing, Indian Institute of Science, Bangalore.

Correspondence: Hardik J. Pandya (hjpandya@iisc.ac.in)

**Supplementary Table 1. Detailed costing for the RapidET system**

| **System component** | **Approximate cost (US$)** |
| --- | --- |
| Microchips (02) | 5.0 |
| Linear guide rails | 70.0 |
| Stepper motors (03) | 100.0 |
| Microcontrollers | 60.0 |
| Machined and 3D-printed parts | 50.0 |
| Miscellaneous electronic components | 35.0 |
| Switched mode power supply | 10.0 |
| Tablet PC | 150.0 |
| **Total** | **480.0** |


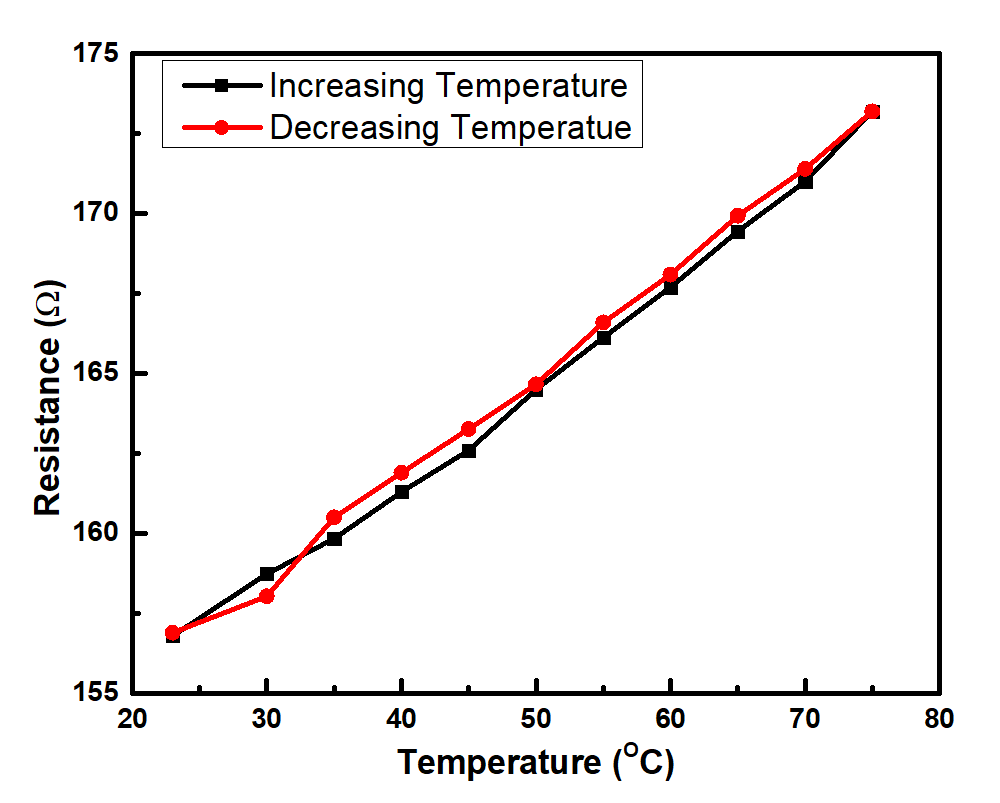


**Fig. S1**. Resistance versus temperature profile of the resistance temperature detectors (RTDs).


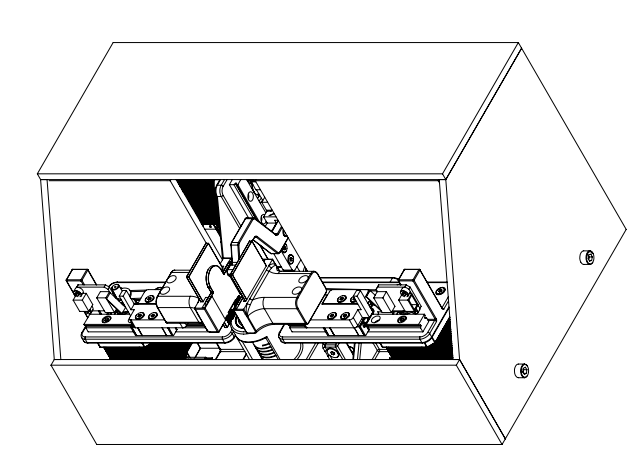

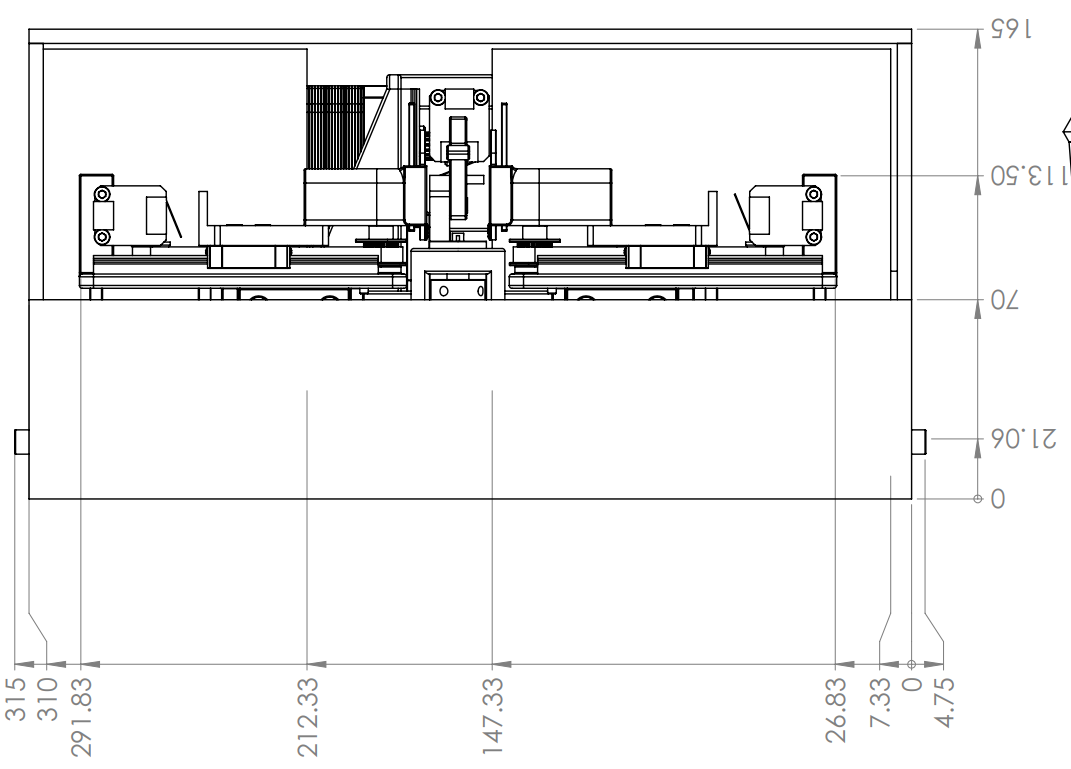

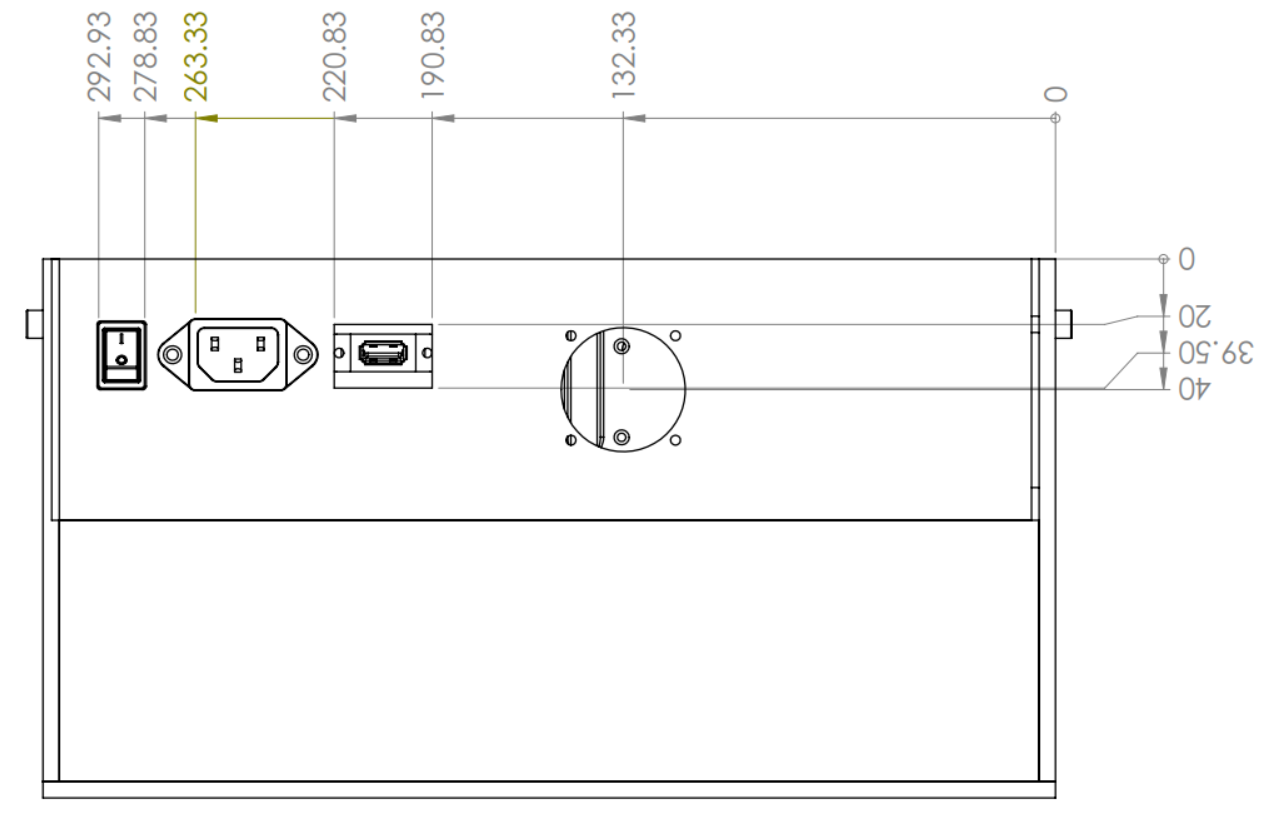


**Fig. S2.** Engineering drawings of the system with the dimensions showing (a) isometric view, (b) front view and (c) connections on the back side.

**(a)**

**(b)**

**(c)**


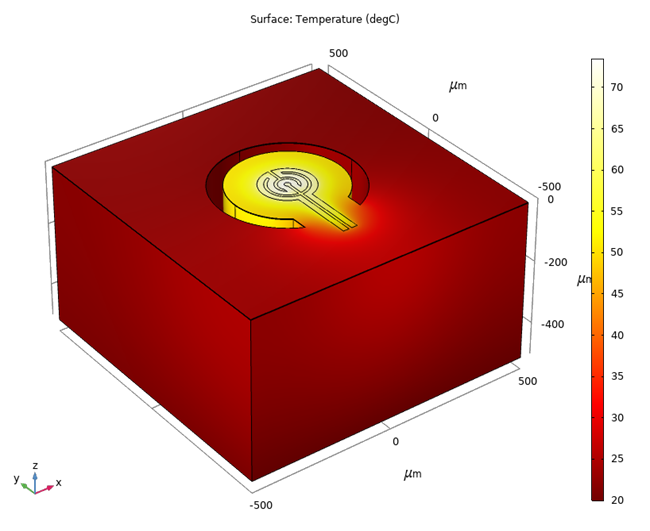

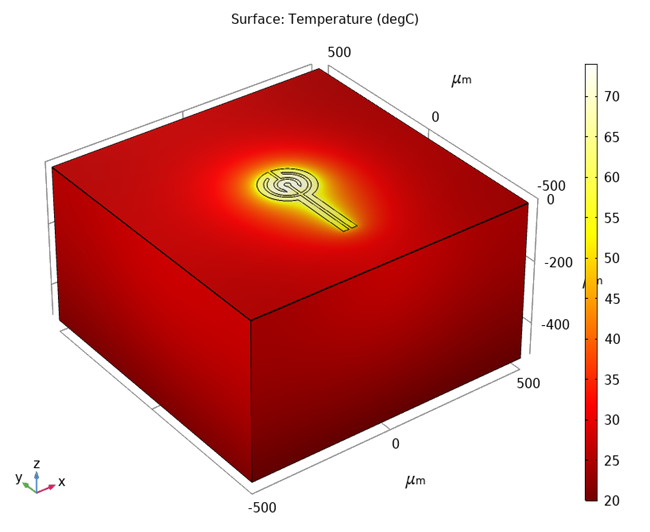

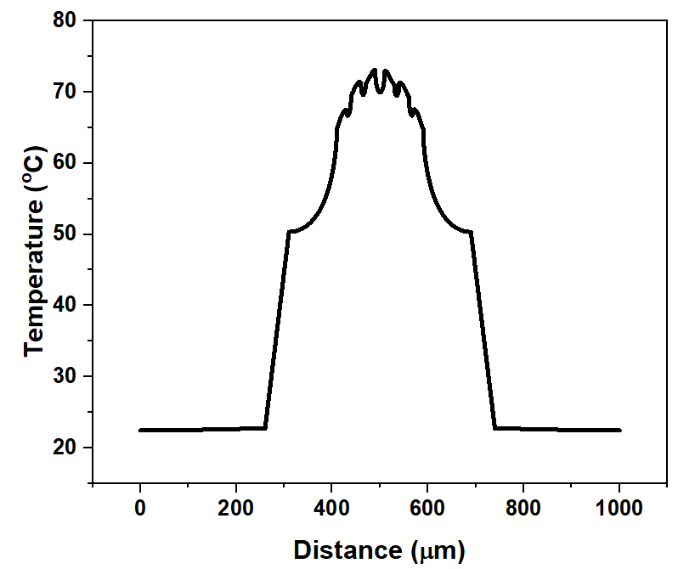

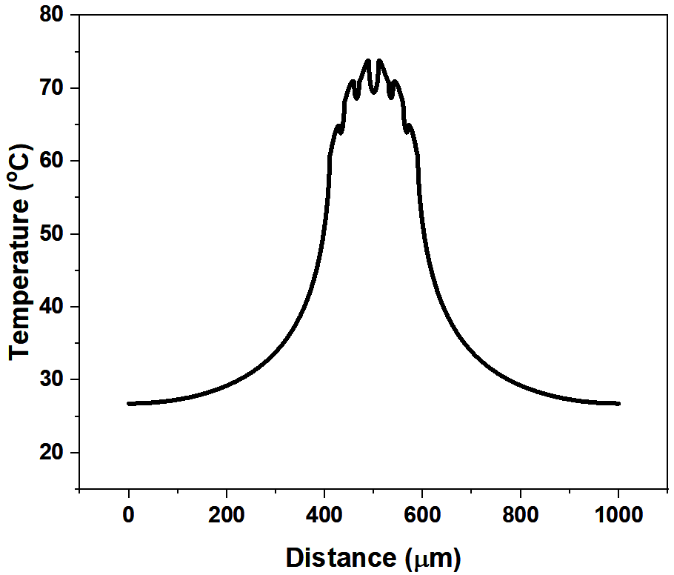


**(a)**

**(b)**

**(c)**

**(d)**

**Fig. S3**. Numerical simulation to show the effect of trench on the thermal profile of the microheater. (a, b) heat map of the thermal profile of the substrate with and without trench, and (c, d) shows the 2D line plot of the temperatures along the axis of the microheater with and without the trench respectively.

**Bulk**

**Microheater**

**Bulk**

**Microheater**

**Bulk**

**Bulk**


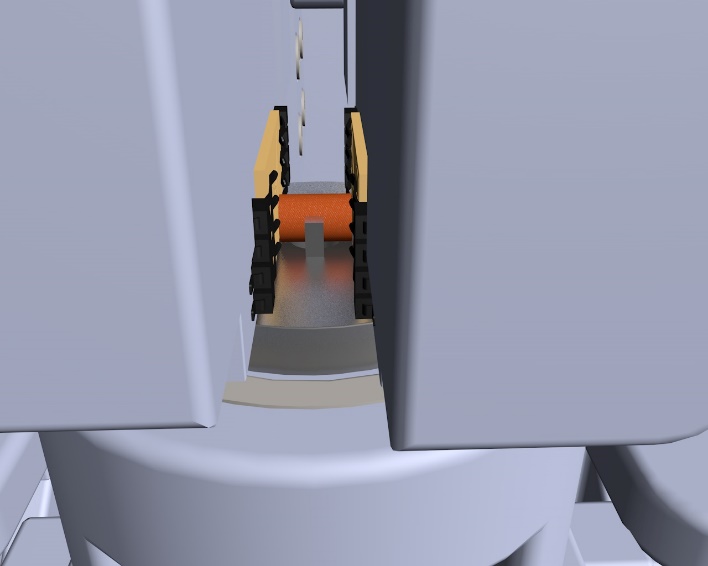

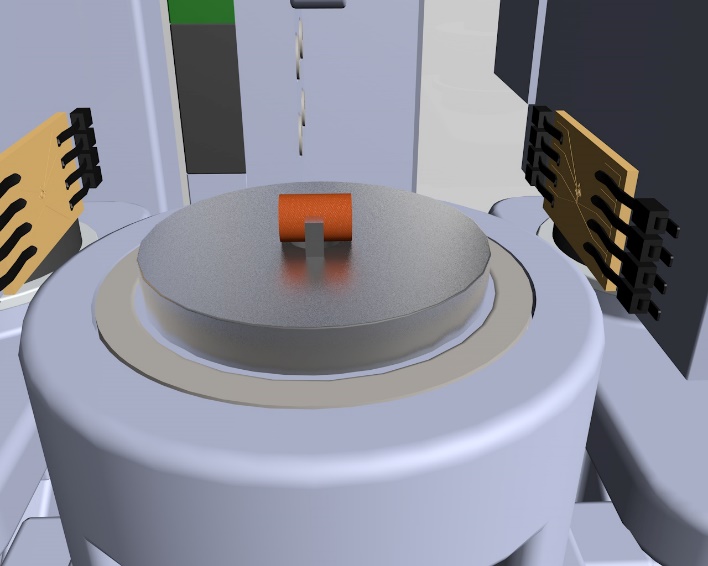


**Fig. S4**. Schematic of measurement test procedure after sample loading on rotary platform. (a) Indenters integrated with microchip and biopsy tissue on sample holder and (b) Indenters in contact with the sample for measurement.

**a**

**b**

**Indenters with microchips in contact with sample for measurement**

**Breast biopsy tissue**

**Indenters with microchips**


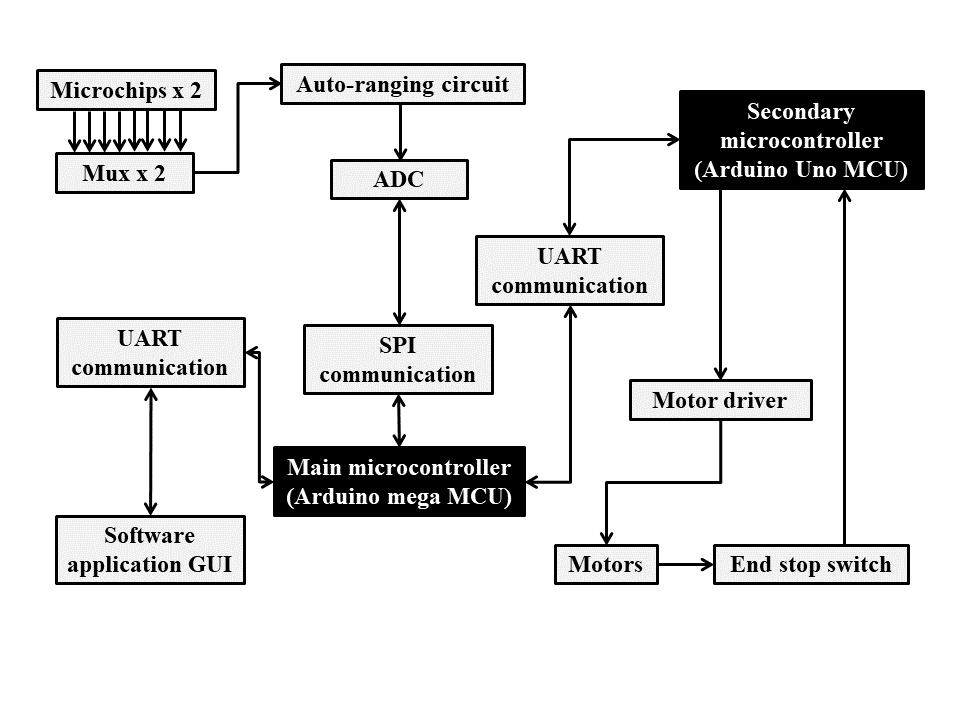


**Fig. S5**. RapidET electronic system architecture showing the major hardware connections, communication protocols, and the primary-secondary arrangement of the microcontrollers.


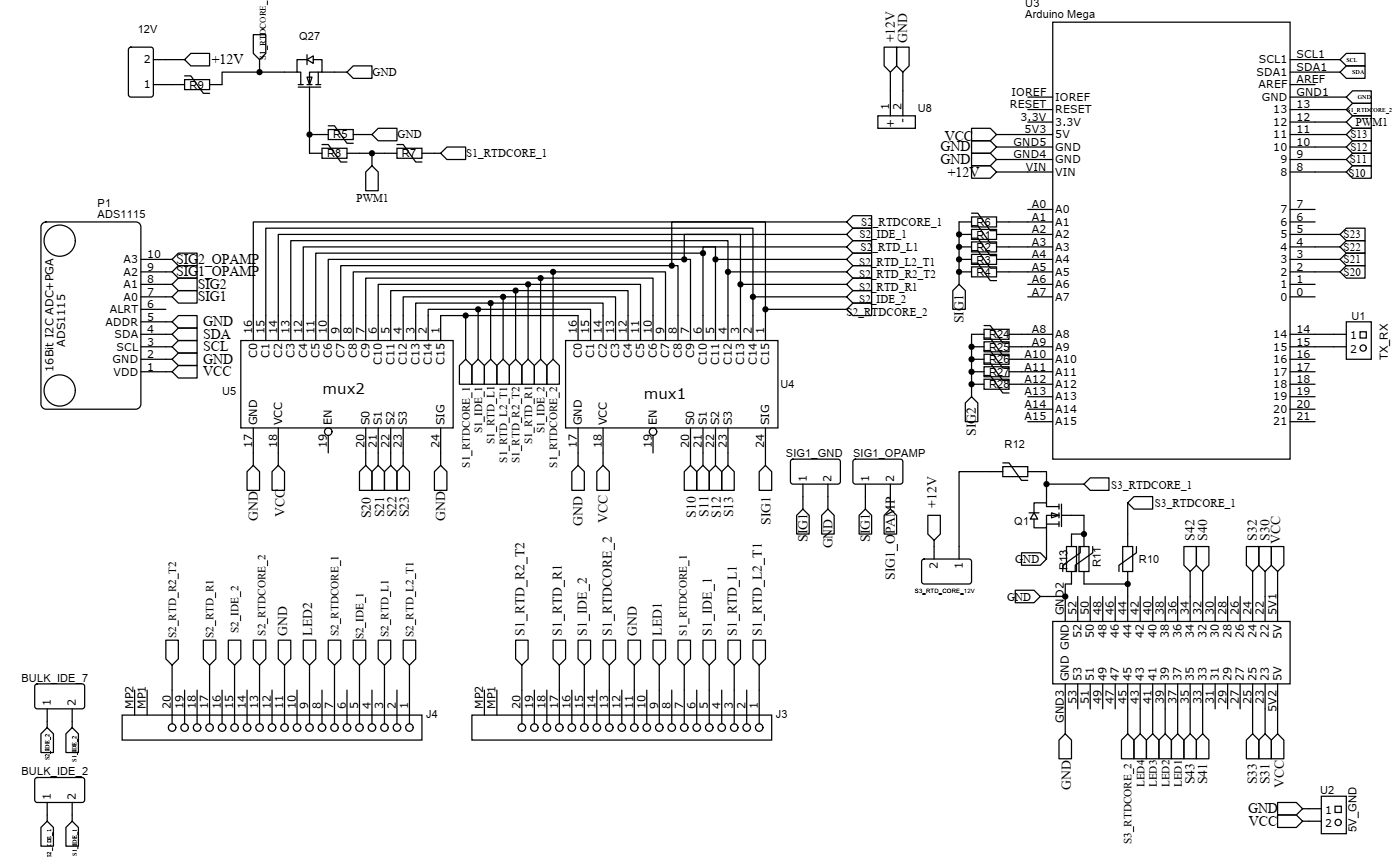


**Fig. S6**. Schematic of the main controller board. (a) Arduino mega controller with the multiplexed sensor input, auto-ranging resistors, driving transistor, supply and ground connection, (b) 16:1 multiplexer modules for measuring different sensor signals, (c) ADS1115 analog to digital converter for converting the values read from the sensor with higher precision, and (d) voltage driver circuit for driving the microheater integrated into the microchip.

**a**

**b**

**c**

**d**


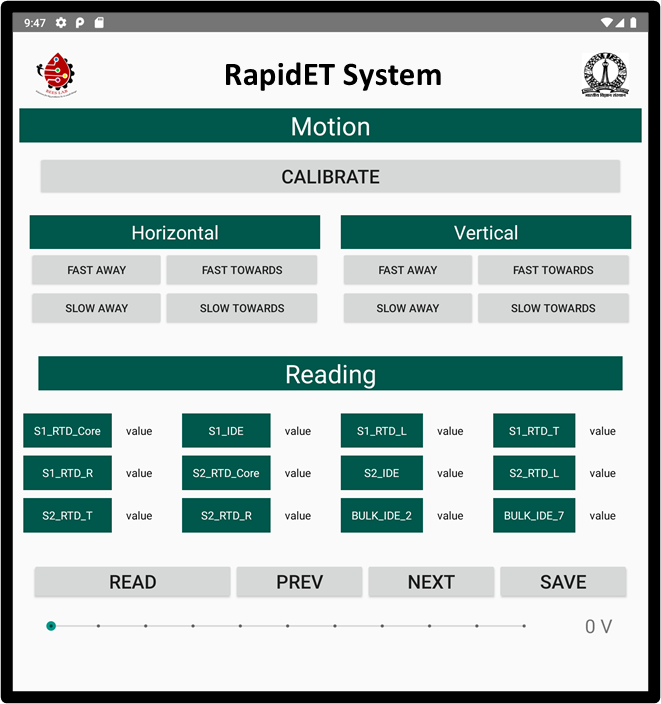


**Fig. S7**. The schematic of the graphical user interface for calibrating, controlling, acquiring, and displaying data from the system.


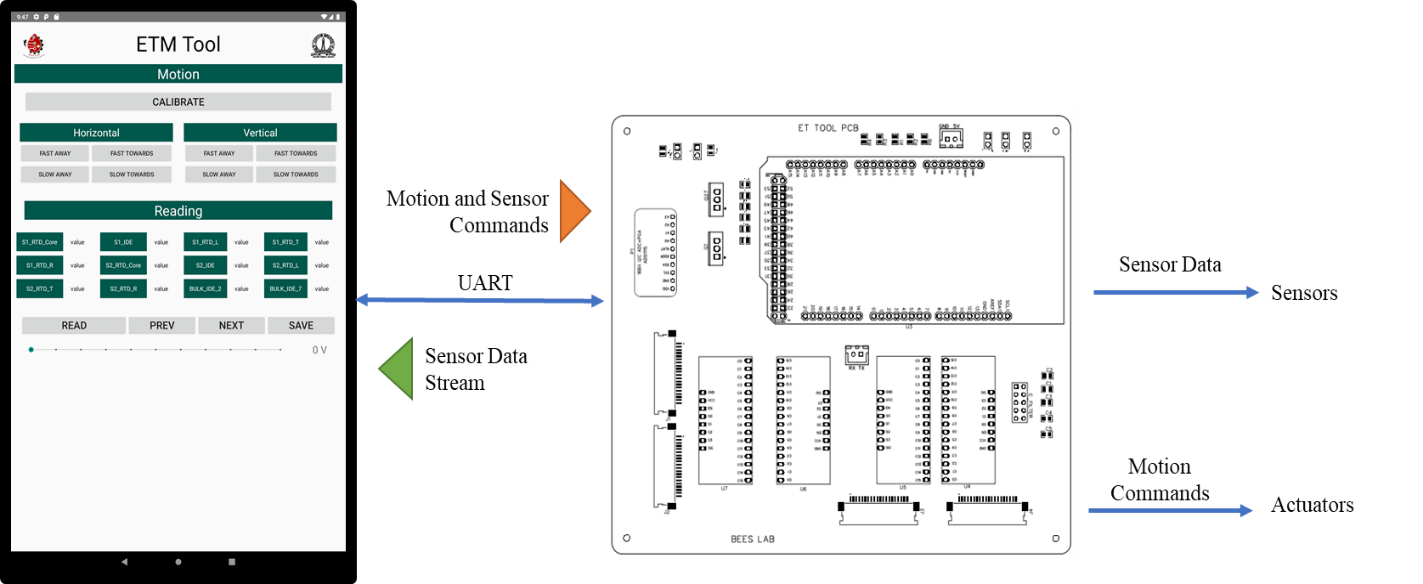

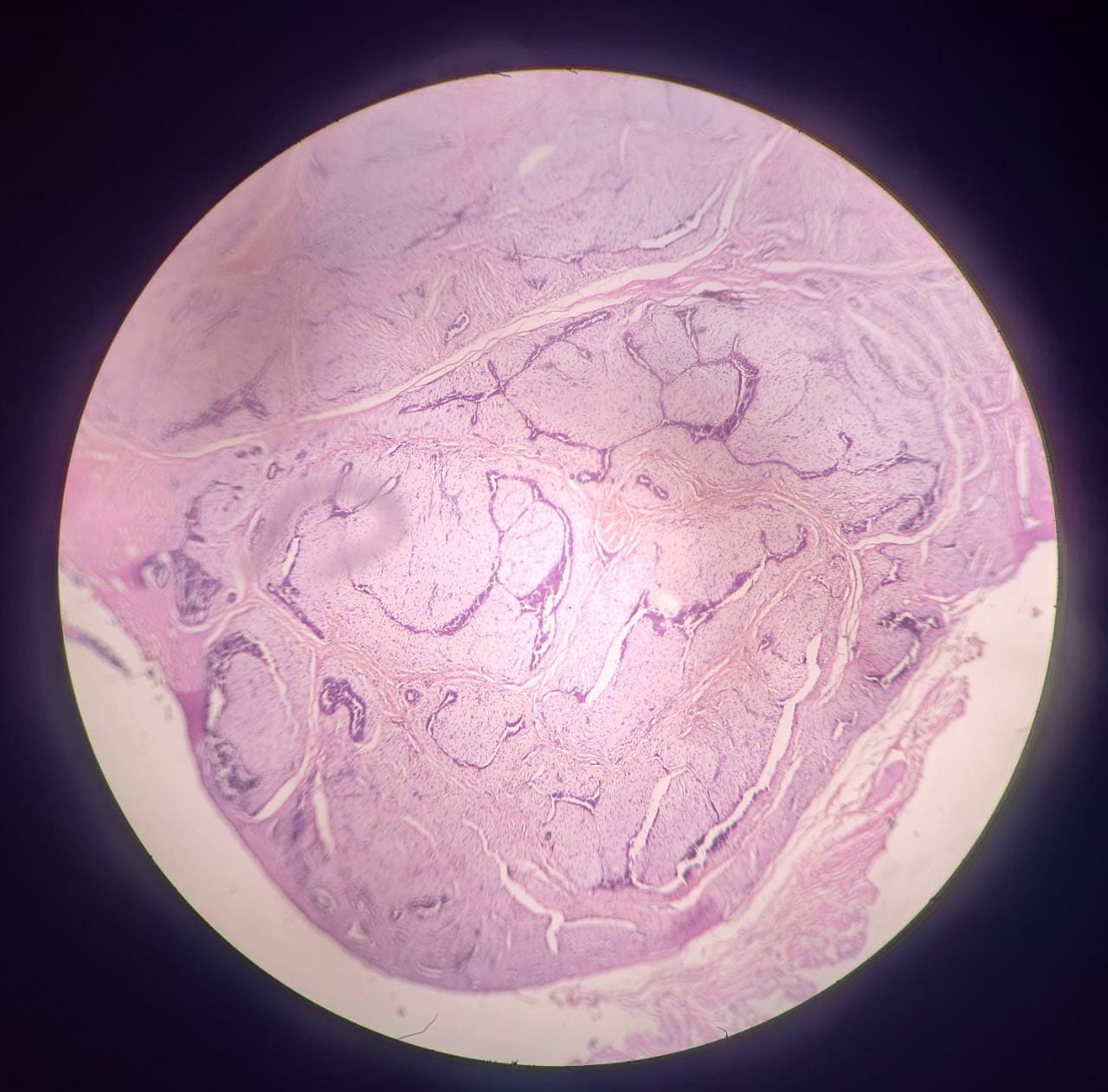

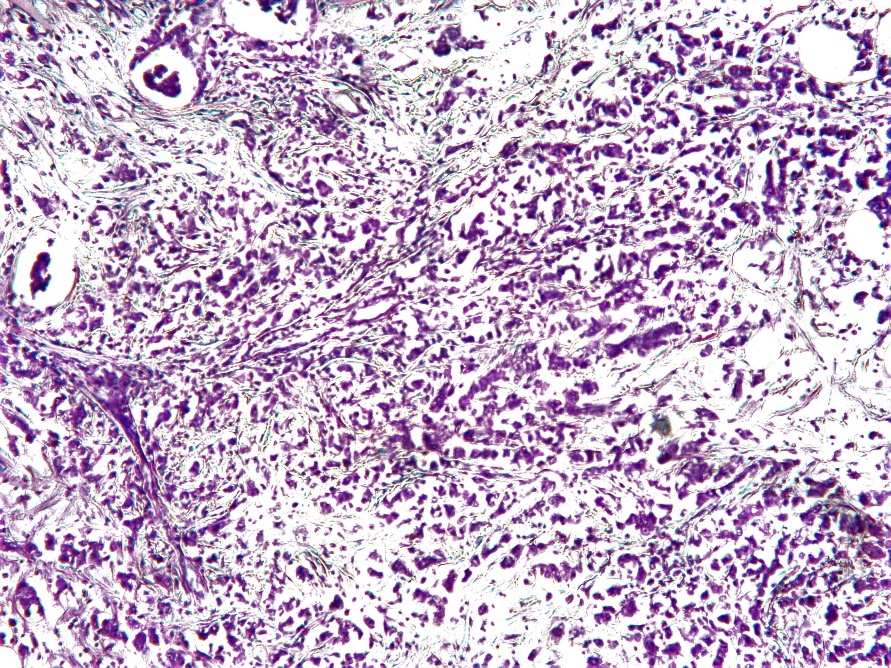


**Fig. S8**. Representative haematoxylin and eosin (H&E) stained histology image of (a) invasive ductal carcinoma (IDC) and (b) fibroadenoma samples.

**a**

**b**

**10 X**

**10 X**
